# Time to Development of Overt Diabetes and Macrovascular and Microvascular Complications Among Patients With Prediabetes: A Retrospective Cohort Study

**DOI:** 10.7759/cureus.20079

**Published:** 2021-12-01

**Authors:** Tyler Finocchio, Satya Surbhi, Charisse Madlock-Brown

**Affiliations:** 1 Department of Pharmacy Services, Yale New Haven Hospital, New Haven, USA; 2 Department of General Internal Medicine, College of Medicine, The University of Tennessee Health Science Center, Memphis, USA; 3 Health Informatics and Information Management, The University of Tennessee Health Science Center, Memphis, USA

**Keywords:** proportional hazards model, survival analysis, diabetes, microvascular, macrovascular, prediabetes

## Abstract

Objective

In this study, we aimed to determine the effect of age, gender, race, and obesity on the development of overt diabetes and macro/microvascular events among patients with prediabetes.

Methods

This was a retrospective cohort study of patient records available through a national electronic health record (EHR) database from 2012 to 2017. Patients with prediabetes in the baseline year of 2012 were identified. Macro/microvascular events were defined as the diagnosis of myocardial infarction (MI), stroke, or chronic kidney disease (CKD). The effects of age, gender, race, and obesity on the incidence of diabetes and macro/microvascular events between 2013-2017 were assessed using the multivariate Cox proportional-hazards model.

Results

Among the total 5,230 patients with prediabetes in 2012, 16.7% developed overt diabetes, and 19.7% developed a macro/microvascular event. Elderly patients (HR: 2.96, 95% CI: 2.12-4.13), males (HR: 1.38, 95% CI: 1.20-1.59), and African-Americans (HR: 1.47, 95% CI: 1.26-1.73) were at a higher risk of experiencing a macro/microvascular event. Additionally, male gender (HR: 1.27, 95% CI: 1.11-1.46) and obesity (HR: 1.24, 95% CI: 1.08-1.43) were significant factors associated with the development of overt diabetes. Furthermore, when diabetes status was added as an interaction term to the Cox proportional-hazards model, no statistical difference was found with respect to any of the other independent variables. It can therefore be inferred that those with prediabetes and overt diabetes had a similar risk of developing macro/microvascular events.

Conclusions

Based on our findings, factors including advanced age, obesity, male gender, and African race significantly impact the progression to diabetes and associated macro/microvascular events.

## Introduction

It is currently estimated that 88 million Americans live with prediabetes or “intermediate hyperglycemia” [1,2]. Prediabetes is characterized by a random fasting blood glucose level of 100-125 mg/dL, a blood glucose level of 140-199 mg/dL two hours after an oral glucose tolerance test, or a hemoglobin A1c (HbA1c) value between 5.7% and 6.4% (39-47 mmol/mol) [3]. Alarmingly, 84.7% of adults with prediabetes are unaware of their condition and the elevated risk of developing diabetes without appropriate intervention [1]. It is estimated that anywhere between 5-10% of those with prediabetes will develop overt diabetes each year. In contrast, another 5-10% may revert to normal blood glucose levels [2]. The risk of developing prediabetes and overt diabetes is substantially higher for those aged 45 years older. There is an increasing trend in younger individuals (18-44 years old) to develop these conditions and the complications related to hyperglycemia [4]. According to the Centers for Disease Control and Prevention (CDC) annual report, the distribution of prediabetes is split almost evenly between the age groups of 18-44, 45-64, and 65+ (28.7, 35.1, and 24.2 million, respectively) [1]. This shows the increased need for the early identification of hyperglycemic conditions among young adults, the importance of timely intervention, and retention in care to prevent disease progression.

In 2018, CDC estimated that 26.9 million Americans were currently diagnosed with diabetes, while another 7.3 million lived with diabetes but were undiagnosed [1,5]. Given the rates of undiagnosed diabetes and associated complications, the need to monitor patients with prediabetes is crucial. Men tend to have a higher prevalence of diabetes compared to women [1]. Also, people of African, Hispanic, and Native American descent have a higher prevalence of developing diabetes than Caucasians [1]. Diabetes is a well-known and modifiable risk factor for various severe and life-threatening complications such as acute coronary syndrome, stroke, lower-extremity amputations, and end-stage renal disease (ESRD) [6]. Adults with diabetes are known to have two to four times higher risk of heart disease compared to those without diabetes, and 68% of patients over the age of 65 die from heart disease while another 16% die from stroke [7]. A high proportion of those with diabetes also has other modifiable risk factors for cardiovascular complications such as obesity, hypertension, and dyslipidemia, which could further elevate the risk and healthcare costs [1]. Due to healthcare costs and lost wages (the wages a person would have received if they had not missed work due to medical issues), diabetes currently costs the United States economy $327 billion annually, and the burden has been increasing over time [1]. Diabetes is a preventable disease, yet the number of adult Americans diagnosed with diabetes continues to rise annually and is expected to reach 39.7 million (13.9%) by 2030 [8]. Therefore, healthcare providers must aim to prevent the growing burden of diabetes and its complications by targeting individuals most at risk of developing diabetes, including those with prediabetes.

Overt diabetes is a risk factor for macrovascular complications (coronary artery disease and stroke) and microvascular complications [chronic kidney disease (CKD), neuropathies, retinopathy]. However, very few people understand the risk of such adverse outcomes among those with prediabetes [9]. In one meta-analysis, adjusted for other cardiovascular risk factors, Levitan et al. found a significant linear relationship between blood glucose levels and overall risk of cardiovascular disease (RR: 1.19, 95% CI: 1.07-1.32). This relationship remained significant after excluding patients with overt diabetes (RR: 1.26, 95% CI: 1.11-1.43). This finding indicates that those without diabetes are still at an increased risk of heart disease based solely on having elevated blood glucose levels [10]. The American Diabetes Association (ADA) has found that patients with prediabetes have a significantly higher number of atherosclerotic plaques in coronary vessels than those with normal HbA1c values. The number and intensity were similar to those with overt diabetes [11]. In a subsequent meta-analysis, Huang et al. found a correlation between prediabetes and an increased risk in a composite of cardiovascular and coronary artery disease, but no difference in stroke or all-cause mortality [12]. In a rebuttal, Vistisen et al. found a statistically significant difference in Kaplan-Meier survival curves for cardiovascular disease and mortality for those with prediabetes diagnosed via HbA1c levels but not for those diagnosed based on fasting blood glucose. This finding indicates that HbA1c is a more predictive diagnosis factor for the overall risk of adverse outcomes [13]. A plethora of data has established prediabetes as an independent risk factor for coronary artery disease, but there is relatively lower data regarding the association between prediabetes and stroke [2]. While overt diabetes is a known risk factor for stroke, there is little information about the risk of stroke among those with prediabetes, but it could be inferred that the risk may be similar to that due to increased glucose levels [14]. Also, those with diabetes tend to have a worse prognosis following a stroke and glucose control does not seem to play a large role in improving outcomes [14]. Since prediabetes is a risk factor for developing overt type 2 diabetes mellitus (T2DM), glucose control in this early stage of hyperglycemia is crucial as it may improve future stroke risk. These studies examined older patients with prediabetes with an average age in the low to mid-60s. The short-term risk of adverse macro/microvascular events for younger patients remains largely undetermined. Additionally, while race, gender, and age were factors listed in baseline demographics, they were not evaluated as risk factors in those studies.

In theory, those with prediabetes who are of older age, male, obese, and those of African, Hispanic, or Native American descent would have an increased risk of developing overt diabetes or experiencing a macro/microvascular event. However, little is known about how these well-known risk factors affect progression to overt diabetes and if prediabetes truly represents a risk of major adverse events as overt diabetes does. Therefore, this study aims to determine how age, gender, race, and obesity affect the development of overt diabetes and the incidence of macro/microvascular events among patients with prediabetes. Additionally, this study explores whether the risk of developing macro/microvascular events is higher for those who develop overt diabetes. To the best of our knowledge, this is the first study to address these questions using a national sample derived from an electronic health record (EHR) system database, which may provide a more accurate representation of population health related to prediabetes management.

## Materials and methods

This was a retrospective study using de-identified and HIPAA-compliant data from the national HealthFacts® database, which is a large vendor-specific database from the Cerner® Corporation. The HealthFacts® database is a repository of anonymous data that includes over 42 million patient records from across the United States for healthcare organizations that use the Cerner Millenium® EHR software [15].

Study population

Adult patients aged 18 years or older with prediabetes were included in the analyses. Patients were classified as having prediabetes if they had at least one HbA1c value between 5.7% and 6.4% (39-47 mmol/mol) during 2012, which served as the baseline year for this study. Since the ADA guidelines recommend that patients with prediabetes receive monitoring at least once a year for the development of T2DM, only those patients with at least one subsequent HbA1c laboratory value during the study period of 2013-2017 were included to determine if the patient developed T2DM [16].

Given the possibility that patients with well-controlled diabetes could also have an HbA1c value between 5.7% and 6.4% (39-47 mmol/mol) during the baseline year, any patient who had a diagnosis of diabetes as per the International Classification of Diseases, ninth revision (ICD-9) during 2012 was excluded. Since metformin is the only medication recommended to prevent T2DM for those with prediabetes, any patient with a prescription for a glucose-lowering medication other than metformin in 2012 was excluded [16]. Any patient with a “null” or “unknown” value for the gender or race fields were also excluded to ensure all independent variables of interest were available for all included patients. One shortcoming of this EHR database was a large degree of heterogeneity and errors related to transcription or the units used to measure height and weight. This problem has been frequently noted in recent literature [17,18]. Due to this limitation and to ensure all included patients had a legitimate body mass index (BMI) value, we restricted the list of acceptable values. Only those with a valid height measured in centimeters and weight measured in kilograms (to calculate BMI) or had BMI readily available within the database were included for the final analysis.

Data collection

Information from the Health Facts® database was imported and a dataset was created within Apache HIVE® data warehouse software (version 3.1.0.3.1.0.0-78). The extracted dataset included information from 2012 through the calendar year of 2017. Therefore, a five-year study period of January 1, 2013, through December 31, 2017, was selected. All baseline demographic information and patient selection was based on the last available values in the 2012 calendar year. Structured query language (SQL) was then used by investigators to extract baseline demographics and other information related to study endpoints. Data pulled from the dataset included patient age, gender, race, BMI, HbA1c values, and dates of those labs, diagnosis of diabetes, hypertension, and hyperlipidemia along with medications used for those conditions, as well as the diagnosis of myocardial infarction (MI), stroke, and CKD including the date of diagnosis. As the information within the database encompassed clinical encounters from 2012 to 2017, both ICD-9 and ICD-10 codes were utilized for the purpose of extracting diagnoses for diabetes, hypertension, hyperlipidemia, MI, non-traumatic stroke (both hemorrhagic and ischemic), and CKD.

Time to development of overt diabetes was defined as the time from the date of prediabetes identification in the baseline period where the last HbA1c value available during 2012 fell between 5.7% and 6.4% (39-47 mmol/mol) and the date of the first diagnosis of T2DM in the follow-up period. Time to macro/microvascular event was defined as the time from the date of prediabetes identification in the baseline period where the last HbA1c value during 2012 fell between 5.7% and 6.4% (39-47 mmol/mol) and the date of the first diagnosis of MI, stroke, or CKD via ICD-9 or ICD-10 codes in the follow-up period. Independent variables were divided into subcategories for the purpose of constructing Kaplan-Meier curves. Based on age, the patients were classified into young (18-44 years), middle-aged (45-64 years), and elderly (65+ years). The race variable was defined as African-American, Caucasian, and other, and due to the low number of patients who identified with a race other than African-American and Caucasian, all other racial categories were combined into the “other” category. BMI was defined according to the standard categories set forth by the CDC, ranging from underweight to class 3 obesity, and patients with a BMI greater than or equal to 30 kg/m^2^ were considered obese [17].

Statistical analysis

Descriptive statistics were reported as proportions or as medians with an interquartile range (IQR). Time to event was assessed via a Kaplan-Meier curve analysis and multivariate Cox proportional-hazards models to examine the differences in the incidence of diabetes as well as the rate of macro/microvascular events by age, gender, race, and obesity. Statistical analysis was performed using the R statistical software (version 3.6.0) including the survival package (version 3.2-3) and the survminer package (version 0.4.7.999). Both Cox proportional-hazards models were adjusted for the development of comorbidities (hypertension, dyslipidemia) and baseline medication therapy. Additionally, in the model assessing the incidence of macro/microvascular events, the development of overt diabetes was added as an interaction term. All tests were two-tailed with an a priori alpha level of 0.05 to be statistically significant.

Ethical approval

Access and use of the Cerner HealthFacts® database were facilitated via an agreement between Cerner® and the University of Tennessee Health Science Center (UTHSC). Use of the HealthFacts® Database for medical research, including this study, is considered exempt from human subjects research by the UTHSC Institutional Review Board as it is a limited dataset according to the HIPAA definition for the de-identification of protected health information [19].

## Results

A total of 158,093 patient records met the criteria of having prediabetes during 2012. However, only 21,795 of those had a follow-up HbA1c laboratory value available during the 2013-2017 study period. Since age, gender, race, and BMI were all variables of interest, only those patients with all four variables available in the database were included for final analysis, which limited the final sample population to 5,230 patients. Baseline demographics information and clinical data are reported in Table 1. The average age of patients was 59.1 years, and the majority of included patients (50.6%) were classified as middle-aged (45-64 years old). Over half of the sample was female (60.8%), and the majority were Caucasian (61.5%). Hypertension and hyperlipidemia were common comorbidities among 69.3% and 59.8% of the sample population, respectively, while medication use for those conditions was low.

**Table 1:** Baseline demographics BMI: body mass index; IQR: interquartile range

Variables		Values
Total population		5,230
Median age in years (IQR)		59.1 (50.4–68.8)
Age category, n (%)		
	Young (18-44 years)	682 (13.0%)
	Middle-aged (45-64 years)	2,648 (50.6%)
	Elderly (65+ years)	1,900 (36.3%)
Gender, n (%)		
	Female	3,178 (60.8%)
	Male	2,052 (39.2%)
Race, n (%)		
	African-American	1,409 (26.9%)
	Caucasian	3,215 (61.5%)
	Other	606 (11.6%)
Median BMI (IQR), kg/m^2^		31.1 (26.7–36.9)
BMI category, n (%)		
	Non-obese (<30 kg/m^2^)	2,284 (43.7%)
	Obese (30 kg/m^2 ^or greater)	2,946 (56.3%)
Median HbA1c (IQR)		
	%	6.0 (5.8–6.1)
	mmol/mol	42 (40–43)
Comorbidities, n (%)		
	Hypertension	3,624 (69.3%)
	Hyperlipidemia	3,125 (59.8%)
Medication use, n (%)		
Angiotensin-converting enzyme (ACE) inhibitor		387 (7.4%)
Angiotensin receptor blocker (ARB)		138 (2.6%)
Calcium channel blocker (CCB)		333 (6.4%)
HMG-CoA reductase inhibitor		524 (10.0%)
Metformin		19 (0.4%)
Thiazide diuretic		183 (3.5%)

Time to development of overt diabetes

**Figure 1:**
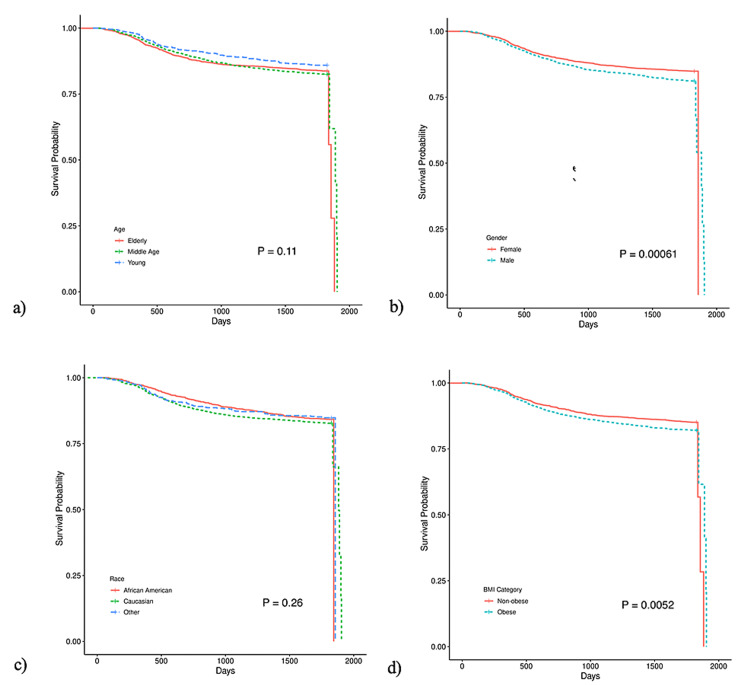
The effect of age, gender, race, and BMI on time to development of overt diabetes BMI: body mass index

The Cox proportional-hazards model and subsequent Kaplan-Meier curves for the effect of age, gender, race, and BMI on time to development of overt diabetes are displayed in Table 2 and Figure 1. Of the total 5,230 patients, 875 (16.7%) were diagnosed with diabetes within the five-year study period. Age and race did not alter the progression to overt diabetes, while patients of male gender and BMI of 30 kg/m^2 ^developed T2DM more quickly (p<0.05) than the rest of the cohort.

**Table 2:** Multivariate Cox proportional-hazards model for time to development of diabetes (days) based on age, gender, race, and obesity* *The model was adjusted for age, gender, race, BMI, baseline HbA1c, and metformin use HR: hazard ratio; CI: confidence interval; BMI: body mass index

Variable	Patients who developed diabetes, n (%)	HR (95% CI)	P-value
Young (18-44 years)	96 (11.0%)	0.82 (0.64–1.03)	0.088
Middle-aged (45-64 years)	467 (53.3%)	1.03 (0.89–1.20)	0.65
Elderly (65+ years)	312 (35.7%)	N/A	N/A
Caucasian	559 (63.9%)	1.08 (0.92–1.27)	0.34
African-American	223 (25.5%)	N/A	N/A
Other	93 (10.6%)	1.00 (0.78–1.27)	0.99
Female	482 (55.1%)	N/A	N/A
Male	393 (44.9%)	1.27 (1.11–1.46)	0.0005
Non-obese (BMI <30 kg/m^2^)	343 (39.2%)	N/A	N/A
Obese (BMI 30 kg/m^2 ^or greater)	532 (60.8%)	1.24 (1.08–1.43)	0.0003

Time to macro/microvascular events (myocardial infarction, stroke, or chronic kidney disease)

**Figure 2:**
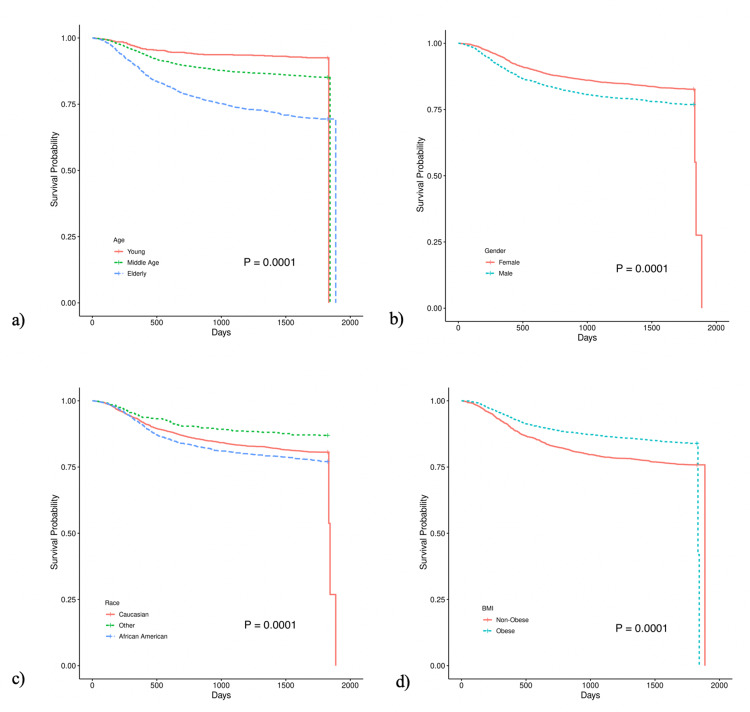
Time to macro/microvascular events (myocardial infarction, stroke, or chronic kidney disease)

Kaplan-Meier curves for the effect of age, gender, race, and BMI on time to the first event for MI, stroke, or CKD are displayed in Figure 2. All variables had a statistically significant effect on the time to macro/microvascular event. Patients aged 65+ years, males, and African-Americans had higher rates of events. Non-obese patients (BMI <30 kg/m^2^), however, had higher rates of MI, stroke, and CKD compared to those who were considered obese.

Risk of macro/microvascular events

The results of the multivariate Cox proportional-hazards model are reported in Table 3. A total of 1,028 patients experienced at least one macro/microvascular event during the study period, and 196 (19%) of the patients who experienced an event also developed overt diabetes; 1,248 events occurred between 2013 and 2017, and 245 (19.6%) of those events occurred in those that developed overt diabetes. Middle-aged and elderly patients experienced a higher proportion of events compared to the young (HR: 1.68, 95% CI: 1.21-2.35; p=0.002 and HR: 2.96, 95% CI: 2.12-4.13; p<0.0001, respectively). The race of the patients was deemed a significant factor as African-Americans had a 47% higher risk to be diagnosed with an MI, stroke, or CKD compared to Caucasians, while those of other races had a 25% lower risk to have such diagnoses. Men were also at a higher risk to experience an event compared to women (HR: 1.38, 95% CI: 1.20-1.59; p<0.0001). Contrary to the initial hypothesis, those with a BMI greater than or equal to 30 kg/m^2^ had a lower risk of experiencing an MI, stroke, or CKD compared to those who were not obese. When diabetes status was added as an interaction term to the Cox proportional-hazards model, none of the variables with overt diabetes were statistically significant.

**Table 3:** Multivariate Cox proportional-hazards model for time to event, including the number of patients who experienced at least one event (MI, stroke, CKD) based on age, gender, race, and BMI categories* *The model was adjusted for age, gender, race, BMI, baseline HbA1c, diabetes status, co-morbidities, and medication use HR: hazard ratio; CI: confidence interval; BMI: body mass index; MI: myocardial infarction; CKD: chronic kidney disease

Variable	Patients with an event, n (%)	HR (95% CI)	P-value
Young (18-44 years)	52 (5.1%)	N/A	N/A
Middle-aged (45-64 years)	394 (38.3%)	1.68 (1.21–2.35)	0.002
Elderly (65+ years)	582 (56.6%)	2.96 (2.12–4.13)	<0.0001
Caucasian	626 (60.9%)	N/A	N/A
African-American	323 (31.4%)	1.47 (1.26–1.73)	<0.0001
Other	79 (7.7%)	0.75 (0.57–0.98)	0.037
Female	554 (53.9%)	N/A	N/A
Male	474 (46.1%)	1.38 (1.20–1.59)	<0.0001
Non-obese (BMI <30 kg/m^2^)	553 (53.8%)	N/A	N/A
Obese (BMI 30 kg/m^2^ or greater)	475 (46.2%)	0.75 (0.63–0.88)	0.0003
Variables with diabetes status added as an interaction term			
Young (18-44 years)	12 (6.1%)	N/A	N/A
Middle-aged (45-64 years)	80 (40.8%)	0.73 (0.37–1.48)	0.3872
Elderly (65+ years)	104 (53.1%)	0.73 (0.34–1.47)	0.3764
Caucasian	112 (57.1%)	N/A	N/A
African-American	65 (33.2%)	1.16 (0.82–1.64)	0.3945
Other	19 (9.7%)	1.28 (0.73–2.25)	0.3825
Female	95 (48.5%)	N/A	N/A
Male	101 (51.5%)	1.15 (0.84–1.57)	0.4007
Non-obese (BMI <30 kg/m^2^)	103 (52.6%)	N/A	N/A
Obese (BMI 30 kg/m^2 ^or greater)	93 (47.4%)	0.94 (0.67–1.30)	0.6905

## Discussion

Diabetes is a well-known factor associated with the increased risk for both macrovascular and microvascular complications, including MI, stroke, and CKD [1]. Patients with prediabetes or intermediate hyperglycemia, however, are also at an increased risk for these complications compared to those considered normoglycemic [10,11]. Prediabetes is often understudied as most patients with this condition are currently undiagnosed and unaware of their risk [1]. The CDC states that age greater than 45 years, race, and obesity are all risk factors for developing both prediabetes and overt diabetes [4]. The purpose of this study was to analyze a national EHR database to identify patients with prediabetes and determine the effect of age, gender, race, and BMI on short-term (five-year) risk of MI, stroke, and CKD among this understudied population. This data-mining investigation also sought to determine time to event and time to diagnosis of overt diabetes.

Many historical studies have stated that people of African, Hispanic, and Native American descent are at a higher risk for the development of diabetes [1,4,20,21].^ ^Recent literature has suggested that race is not a direct factor in the development of diabetes but that any disparity in diabetes prevalence between races could be attributed to other factors such as socioeconomic status, obesity, and family history of diabetes [22,23,24]. This study corroborates this finding in that once a patient was diagnosed with prediabetes, progression to overt diabetes was similar between African-Americans, Caucasians, and those of other races. This also correlates with the findings of Dagogo-Jack et al., who studied racial disparity among normoglycemic offsprings of parents with T2DM and found no difference in the development of prediabetes or the progression to overt T2DM between African-Americans and Caucasians based on race. Instead, only age, male gender, and factors associated with obesity (i.e., increased weight, BMI, waist circumference, total fat mass, and trunk fat mass) were significant predictive variables for the development of prediabetes/diabetes [25]. In this analysis of patients with prediabetes, no difference was found in the progression to overt diabetes based on the age categories defined in this study. However, both male gender and obesity were found to be significant factors that increased the risk of disease progression to T2DM.

One interesting finding of this data-mining investigation was the low rate of adherence to the recommendations set forth by the ADA for the monitoring and treatment of patients with prediabetes. The ADA guidelines for the care of patients with diabetes in 2012 and each revision since have recommended that those with prediabetes should have their HbA1c measured on at least an annual basis to monitor the development of T2DM [16]. While 158,093 patients were identified to have prediabetes in 2012, only 21,795 (13.8%) had at least one subsequent HbA1c level measurement between 2013 and 2017. This gap in monitoring is a measurable goal for diabetes prevention programs that could potentially improve rates of disease progression and adverse outcomes. Treatment recommendations for those with prediabetes center around weight loss through diet and exercise, but the ADA guidelines also recommend the use of metformin in those with prediabetes for the prevention of T2DM, especially those with BMI >35 kg/m^2^, age <60 years, or a history of gestational diabetes [16]. The use of metformin for the prevention of T2DM was extremely low in this study cohort as only 19 (0.4%) patients with prediabetes were prescribed metformin. When diet and exercise were compared to metformin for the prevention of T2DM, behavioral modification reduced the incidence of diabetes by 58% compared to only a 31% reduction for those who took metformin as opposed to placebo [26]. It was determined that seven people would be treated with a lifestyle intervention program, compared to 14 people who would need to take metformin to prevent one diagnosis of diabetes in a three-year period [26]. Given these numbers, if all 5,230 patients were prescribed metformin, 373 diabetes diagnoses (42.6%) could potentially have been prevented.

Age, gender, race, and BMI all had a significant effect on the diagnosis and time to event for the composite of MI, stroke, or CKD. As expected, middle-aged and elderly patients had a higher risk of events compared to young individuals, which is likely attributed to an increased risk of comorbidities in addition to prediabetes. There is a large amount of heterogeneity in the published literature regarding the effects of gender and race on the prevalence and mortality related to MI, stroke, and CKD, but this study found that men and people of African descent had higher rates of events. In one systematic review, it was reported that Hispanics and Asians had a lower prevalence of stroke and cardiovascular disease compared to non-Hispanic white and non-Hispanic blacks with T2DM. Mortality related to heart disease or stroke, however, was higher among Hispanics and non-Hispanic blacks [27]. Since only a small number of patients identified with a race other than African-American or Caucasian, all other race options were compiled in the “other” category. The results of this study are in line with the findings of the above-mentioned systematic review in that those of African descent had higher rates of events compared to Caucasians while those of other races had a lower risk.

Obesity is a well-established risk factor for cardiovascular disease and other adverse clinical outcomes [7].^ ^Contrary to that fact, an unexpected finding of this study was a higher rate of MI, stroke, and CKD among non-obese individuals (BMI <30 kg/m^2^). Further investigation would be needed in order to determine the root cause of this finding. One possible explanation could be due to a notable age difference between the groups, as individuals with BMI <30 kg/m^2^ were slightly older than those with BMI >30 kg/m^2^, with an average age of 63 vs. 57 years, respectively. Even after adjusting for age and other factors, we found that the non-obese group had a higher risk for macro/microvascular events. This finding could be confounded by other factors that were not measured in the study. Persons of advanced age also have an increased risk of other possible comorbidities, which were not included in this analysis model and may have contributed to a greater number of events [28]. When the diabetes status was added as an interaction term within the Cox proportional-hazards model, age, gender, race, and BMI were no longer statistically significant. It can then be inferred that the risk of macro/microvascular events is similar for patients with prediabetes as it is for patients with overt diabetes. This supports the data in previous literature by indicating that those with prediabetes and overt diabetes would have a similar rate of events when corrected for other factors. In one study that assessed risk factors for macrovascular events among patients with T2DM, age, gender, uncontrolled diabetes, and hypertension were all significantly associated with increased risk of MI and stroke [29]. A key finding of this investigation was that the majority of those with prediabetes also had a diagnosis for hypertension and hyperlipidemia (69.3 and 59.8%, respectively), and yet only a small percentage of patients were prescribed first-line antihypertensive or lipid-lowering medications (Table 1). Appropriate treatment of these comorbidities could decrease the rate of events and disease progression, and therefore should be incorporated in diabetes prevention programs.

Strengths and limitations

A major strength of the study is that it was a multivariant analysis that corrected for a variety of variables associated with the risk of macro/microvascular complications, and it had a relatively large sample size. To our knowledge, this is also the first study to evaluate the effect of age, gender, race, and BMI on time to development of overt diabetes and time to MI, stroke, and CKD among patients with prediabetes. Limitations of this analysis include the low rate of follow-up HbA1c values, which significantly reduced the number of potential patients included in the final analysis. Some patients could have been lost to follow-up or perhaps a high portion of patients were also seeing providers that did not utilize Cerner® as an EHR vendor. Therefore, some records could have been missing from each patient’s profile. Additionally, study endpoints were limited to major adverse complications related to diabetes and did not assess rates of other known microvascular complications such as retinopathy or neuropathy. Another limitation was the heterogeneity related to errors in transcription or units of measurement in documented height and weight, which greatly reduced the number of patients included in the study due to the inability to calculate a valid BMI. There was also a lower number of patients who identified with a race other than African-American or Caucasian, which limited the generalizable or extrapolatable validity of this study’s results to other racial minorities. Future research opportunities exist in examining what factors led to the higher rate of MI, stroke, and CKD among non-obese individuals, and on the impact of prevention programs targeting those with prediabetes, as well as medication therapy management for diabetes and comorbidities.

## Conclusions

Among patients with prediabetes, male gender and obesity were associated with an increased rate of developing overt diabetes. Age, gender, race, and BMI were all significant factors associated with the diagnosis of MI, stroke, and CKD. Additionally, patients who developed diabetes had a similar risk of such events when compared to patients with prediabetes. This investigation found a low rate of medication use, both in terms of metformin use for the prevention of T2DM and the use of medication therapy for hypertension and hyperlipidemia. The data presented are hypothesis-generating and deserves a more in-depth investigation as there are several factors associated with the risk of MI, stroke, CKD, and the development of diabetes.
